# Assessing the antigenicity of different VP3 regions of infectious bursal disease virus in chickens from South Brazil

**DOI:** 10.1186/s12917-021-02956-0

**Published:** 2021-07-30

**Authors:** Ana Paula Gori Palka, Tatiana Reichert Assunção de Matos, Claudemir de Souza, Danilo Santos Eugênio, Marco Aurélio Krieger, Stenio Perdigão Fragoso, Daniela Parada Pavoni

**Affiliations:** 1 Instituto Carlos Chagas/ICC, Fundação Oswaldo Cruz/Fiocruz, Curitiba, PR Brazil; 2 Instituto de Tecnologia do Paraná/Tecpar, Curitiba, PR Brazil; 3 Programa de Pós-graduação em Biologia Celular e Molecular, Universidade Federal do Paraná/UFPR, Curitiba, PR Brazil; 4 Programa de Pós-graduação em Biociências e Biotecnologia, Instituto Carlos Chagas/ICC, Fundação Oswaldo Cruz/Fiocruz, Curitiba, PR Brazil

**Keywords:** Infectious bursal disease, Gumboro disease, VP3, Antigenic regions, ELISA

## Abstract

**Background:**

Infectious bursal disease (IBD), also known as Gumboro disease, is a viral infection that causes mortality and immunosuppression in chickens (*Gallus gallus*). VP2 and VP3 are the major structural viral capsid components and are the most immunogenic proteins of IBD virus (IBDV). Reliable diagnostic tests using VP2 and VP3 produced in heterologous systems are important tools to control this infection. One advantage of an IBD diagnostic based on VP3, over those that use VP2, is that VP3 has linear epitopes, enabling its production in bacteria.

**Results:**

We tested the suitability of recombinant VP3 (rVP3) as a diagnostic reagent in an enzyme-linked immunosorbent assay (ELISA). Compared with a commercial test, rVP3 ELISA showed high sensitivity and specificity as a diagnostic tool for vaccinated animals. In addition, rVP3, but not the commercial ELISA, was able to detect antibodies in nonvaccinated chickens, probably developed against circulating IBDV strains. It was possible the assessment of VP3 regions antigenicity using chicken antisera.

**Conclusions:**

The full-length recombinant VP3 can be used to assess post vaccination immunological status of chickens and its production is feasible and inexpensive. The evaluation of VP3 regions as candidates for general use in the diagnosis of IBD in chickens should be conducted with caution. Our work was the first to identify several regions of VP3 recognized by chicken antibodies.

**Supplementary Information:**

The online version contains supplementary material available at 10.1186/s12917-021-02956-0.

## Background

Infectious bursal disease (IBD), also known as Gumboro disease, is an important contagious condition associated with immunosuppression in young chickens by the destruction of the precursors of antibody-producing B cells. In few-week-old chickens (3–6 weeks), the atrophy of the bursa of Fabricius predisposes them to several diseases and interferes in the responses to vaccination against other pathogens and may culminate in death [1–6].

This disease is caused by IBD virus (IBDV), a member of the Birnaviridae family, an icosahedral nonenveloped particle with a diameter of about 65 nm. Its genome is composed of two segments of double-stranded RNA. VP2 and VP3, the major structural viral capsid components, are encoded by the largest RNA segment, with approximately 3,300 base pairs (bp), and are the most immunogenic proteins of IBDV [7–12].

VP2 elicits a very strong immune response, and the neutralizing antibodies recognizes conformational epitopes located in the central hypervariable region [11, 13, 14]. VP3 response is not neutralizing but is the first to be detected [9, 15].

The diagnosis of IBD is made by the clinical examination of symptoms, histopathological examination of bursae, virus isolation, virus detection by polymerase chain reaction (PCR) and immunological tests for detection of virus or antibodies, such as ELISAs, which are currently the most used diagnostic test for IBD because of their high sensitivity, specificity and feasibility [16]. There are several commercial ELISA kits for the detection of antibodies against IBDV, and most of them use the whole virion as the antigen. The substitution of the whole virion by recombinant proteins produced in heterologous systems or by synthetic peptides is a trend in the diagnostic field due to the biological safety, ease of production and cost reduction [17–22].

Diagnostic assays based on VP3 have advantages over those based on VP2 protein. The main VP2 antigenic region is a conformational epitope whose structure is solely preserved in high-cost eukaryotic expression systems. In contrast, VP3, a more conserved target for the immune response than VP2, has linear epitopes, enabling its production in bacteria, an economical and easier expression system [20, 22–24].

One of the concerns related to diagnostics in countries where the epidemiology is not well studied is the fact that commercial kits used to assess immunological status are developed using strains that circulate in other areas of the globe. In Brazil, huge producers vaccinate poultry with commercial vaccines based on circulating strains in North America and Europe, and the evaluation of the immune response is performed with kits with sensitivities and/or specificities suitable for the vaccine strains as antigens, or at least very similar ones.

However, the dissemination of domestic breeders, without the same sanitary controls imposed on huge commercial producers and aiming to have animal production free of medicines, propagates a reservoir for pathogens and is a concern for Brazilian governmental agencies. Thus, it is urgent to evaluate the sanitary conditions of those stocks. In these cases, the use of commercial devices for pathogen diagnosis is only valid if the generated antibodies against the circulating strains can recognize the antigens used in those kits. The customization of diagnostic methodologies requires the genetic characterization of the circulating strains, whose variability is little known. Few works report the genetic variability of IBDV in Brazil [25–34].

Although the VP3 sequence is conserved, *in silico* analysis shows some variability in its amino acid sequence [23]. Hence, it is important to analyze the antigenic determinants of the protein to conceive a universal diagnostic assay for IBDV. Additionally, the genetic background of the animals used to provide the antibodies for the trial should be considered, as it can influence the recognized epitopes of VP3. In fact, the described VP3 epitopes were identified using preferably monoclonal antibodies produced in mice [23, 35–38]. In this work, we have designed several constructs of VP3 for recombinant production in bacteria. We evaluated these antigens by ELISA to detect antibodies against VP3 in antisera from vaccinated and nonvaccinated chickens maintained by domestic producers and to map VP3 epitopes using chicken antisera.

## Results

### Expression of recombinant VP3 and its fragments

In this study, a full-length rVP3 protein and several overlapping VP3 fragments were produced to assess the antigenicity of the protein and to map antigenic regions using antisera obtained from chickens maintained by Brazilian producers. The complete VP3 sequence expressed corresponds to amino acids 724 to 1012 of the polyprotein precursor of the attenuated vaccine strain ABI52864.1 [39]. Even though VP3 is a conserved protein among isolates, analysis of several sequences around the world shows amino acid substitutions that can be relevant to the recognition by antibodies.

**Fig. 1:**
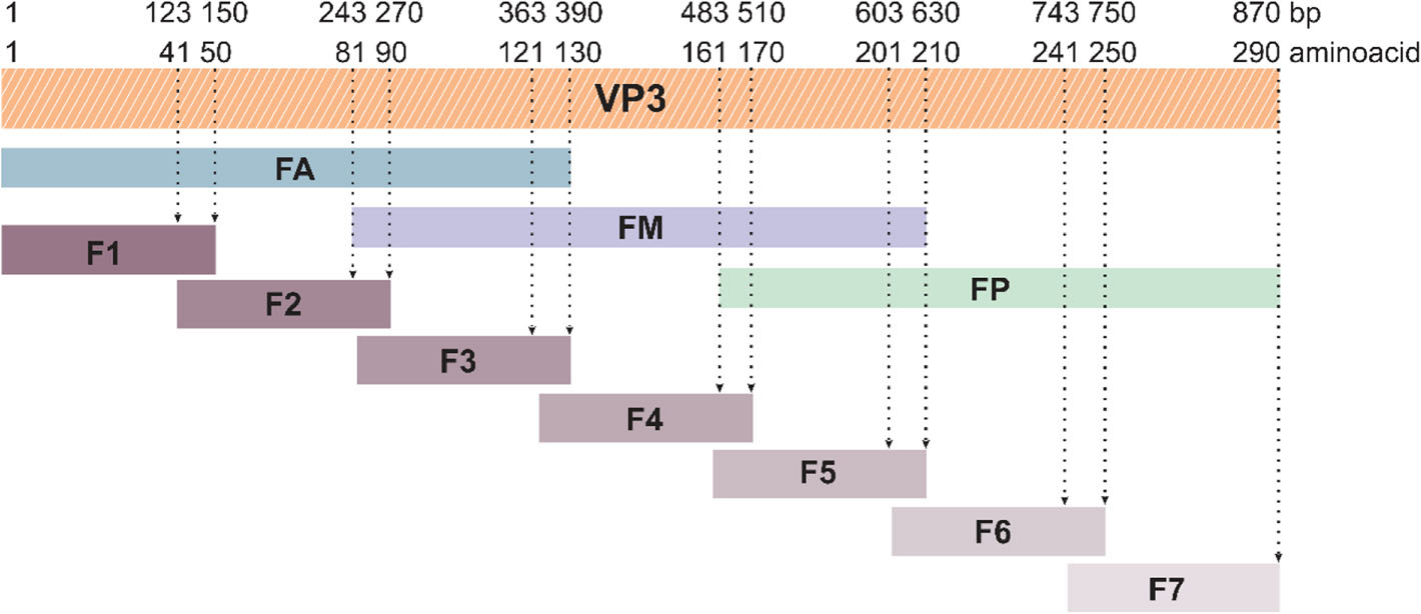
Schematic representation of VP3 fragments. Overlapping regions from the VP3 nucleotide sequence were PCR-amplified to generate three fragments of 390 bp (FA, FM and FP, 130 amino acids) and seven fragments of 150 bp (F1 to F7, 50 amino acids)

**Fig. 2:**
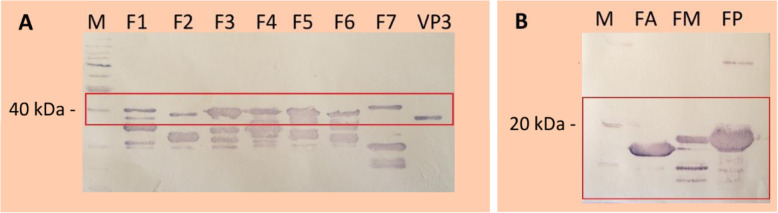
Western blot of rVP3 and purified fragments using anti-6×His monoclonal antibody. (**A**): Fragments F1, F2, F3, F4, F5, F6, F7 fused to GFP and recombinant VP3. (**B**): Fragments FA, FM, FP. M: Molecular marker. The region of the expected full-length fragment is highlighted by rectangles

Recombinant VP3 was produced by *Escherichia coli* as inclusion bodies and solubilized with 2 M urea (yielding approximately 150 mg of purified protein/liter of culture). Three larger VP3 fragments (named as FA, FM and FP) of 130 amino acids (approximately 18 kDa), overlapping each other by 50 amino acids, were expressed in *E. coli*, as well as seven shorter fragments (F1 to F7) consisting of 50 amino acids, overlapping each other by 10 amino acids (Fig. 1). The shorter fragments were expressed in fusion with green fluorescent protein (GFP) to increase their molecular weight to approximately 37 kDa and to visually monitor the protein expression (Fig. 2). All fragments could be recovered from the soluble fraction.

### Recombinant VP3 ELISA can be used to detect anti-IBDV antibodies

We evaluated whether the rVP3 could be used for the diagnosis of IBD by ELISA. First, we tested antisera from the 79 vaccinated animals (adult chickens or chicks whose mothers were vaccinated) with a commercial IBD ELISA (IDEXX Laboratories). All the samples were positive. Then, a subset of eight chicken antisera was used to determine the optimal conditions of the rVP3 ELISA, regarding the antigen amount to sensitize the plate, secondary antibody dilution and antisera sample amount (Additional file 1). After this optimization, the subsequent tests with our rVP3 ELISA were performed with 0.5 µg/ml antigen, a 1:40,000 dilution of secondary antibody and a 1:500 dilution of antisera. The rVP3 ELISA, similar to the commercial ELISA, also gave positive results for all 79 samples of vaccinated adult chickens and chicks whose mothers were vaccinated. Additionally, the rVP3 ELISA did not detect antibodies in the antisera of 19 free-IBDV animals. The receiver operating characteristic (ROC) analysis indicated an optimal cut-off of 0.0496, resulting in sensitivity and specificity values of 100 %. The correlation of the optical density (O.D.) values between the two tests was low (*R*^2^  =  0.24) (Additional file 2).

Additionally, we investigated the ability of the rVP3 ELISA to detect the presence of antibodies developed against a wild type virus strain. For that assessment, the antisera of seven nonvaccinated chickens and two chickens with unknown immunization status obtained from domestic producers were tested with our rVP3 ELISA and the commercial ELISA. All the samples were considered negative by the commercial ELISA, except one. In contrast, all antisera were reactive with the rVP3 ELISA.

### Evaluation of VP3 regions antigenicity

**Fig. 3:**
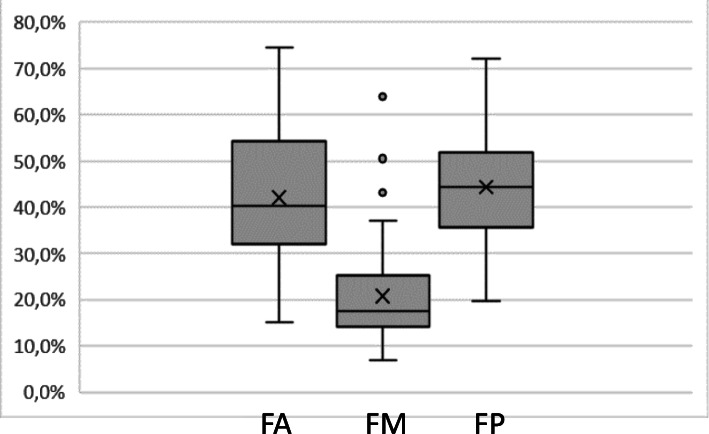
Evaluation of the reactivity of the VP3 fragments FA, FM and FP. Y-axis corresponds to signal of the fragment as the percentage of rVP3 O.D. values. The box delimitates 25-75 % percentiles; line within the box marks the median; x within the box marks the mean; whiskers above and below the box mark 100 and 0 % percentiles; dots mark outliers

We further investigated the antigenicity of distinct regions of VP3. Thirty-nine antisera samples (positive by the commercial and rVP3 assays) were tested with an ELISA sensitized with three regions of VP3 (FA, FM and FP). The three fragments were recognized by all the samples, though the FM fragment presented lower O.D. values (Fig. 3). Additional file 3 displays the O.D. values of each sample.

To accurately map the epitopes, shorter fragments, each composed of 50 amino acids, were analyzed by ELISA (Table 1). Considering that there is no positive control to be used to define the cutoff, the result of each fragment signal is presented as a percentage of the rVP3 signal. Fragment signals above 10 % are shown in Table 1. All O.D. values can be found in Additional file 4.

**Table 1:** Evaluation of VP3 fragments F1 to F7 by ELISA

Sera	rVP3	F1	F2	F3	F4	F5	F6	F7
ME223	0.199	(13.8)	43.0	-	-	-	-	28.6
ME186	0.201	-	(15.2)	-	-	-	(15.2)	(14.7)
G27	0.212	(12.3)	73.3	-	(15.6)	(16.7)	-	41.5
G17	0.236	-	56.6	-	-	-	-	57.4
G43	0.315	-	52.4	-	(15.4)	(10.6)	-	48.7
G12	0.315	-	44.9	-	-	-	-	67.3
ME33	0.328	-	34.8	-	-	-	-	36.4
S9	0.358	-	35.1	(11.7)	20.4	-	-	35.3
ME93	0,384	16.9	27.0	-	-	23.7	19.1	42.7
G16	0.409	-	18.8	-	-	-	-	44.4
ME208	0.447	-	40.5	-	-	-	-	63.4
G33	0.632	-	-	-	-	14.9	-	19.0
G1	0.761	-	22.5	-	-	-	-	150.8
G25	0.836	-	10.9	-	-	-	-	37.7
G34	0.880	-	10.1	-	-	-	-	217.0
G18	0.984	-	16.5	-	-	13.8	-	33.3
G11	1.021	-	19.2	-	-	18.9	-	29.1
NC	1.034	-	-	-	-	27.7	-	-
G9	1.062	-	17.1	-	-	-	-	18.7
ME228	1.069	-	-	-	-	-	-	-
G23	1.095	-	11.6	-	-	35.0	-	19.0
G20	1.143	-	19.9	-	-	11.2	-	17.5
ME211	1.200	-	15.8	-	-	-	11.1	14.2
G22	1.203	-	15.5	-	-	16.2	13.3	25.9
G28	1.226	-	13.9	-	-	34.0	-	17.9
ME219	1.298	-	-	-	-	13.9	47.3	-
ME178	1.386	-	17.9	-	-	57.9	-	61.9
ME214	1.409	-	16.2	-	-	-	10.8	13.4
G14	1.453	-	20.9	-	-	44.4	13.9	36.1
ME227	1.478	-	11.7	-	-	14.5	14.5	-
G6	1.502	-	-	-	-	22.8	-	-
ME267	1.524	-	15.8	-	-	23.9	21.6	21.2
G42	1.824	-	-	-	-	-	-	49.0
ME262	1.990	-	----	-	25.2	45.9	32.5	12.8
ME88	2.205	----	18.1	-	-	26.7	15.5	59.1
ME86	2.888	-	----	-	11.7	13.6	78.9	21.2
ME275	3.065	-	-	-	-	36.3	-	-
ME39	3.067	-	----	-	----	21.6	88.2	51.1
G47	3.535	-	-	-	-	15.4	-	111.1

The fragment signal is presented as a percentage of the rVP3 signal. Only values above 10 % are reported. Fragment signals which are above 10 % of the rVP3 signal but showing O.D. values below 0.050 are indicated in parenthesis. Fragment signals which are below 10 % of the rVP3 signal but showing O.D. values above 0.150 are indicated with “----”. These values possibly reflect a true positive recognition. The sera samples are listed in ascending order of the rVP3 O.D. values. *ME* vaccinated chickens; *G* one-day-old chicks; *S9* nonvaccinated chicken; *NC* unknown vaccination status

With few exceptions, the fragments did not exhibit absorbance as high as the rVP3. Some distortions may be occurring since fragment values were the result of subtracting the GFP value from the chimera O.D. value (the fragment is fused to GFP). In addition, the fragments were soluble and the rVP3 protein was expressed in inclusion bodies hence the fragments might be being expressed in a conformation different from that acquired in the context of the full-length protein. Therefore, different antibodies may be binding to the rVP3 and to the fragments. Anyway, even with lower O.D. values, the fragments could still be informative.

Most of the antisera samples recognized VP3 fragments F2 and F7, corresponding to amino acid 41 to 90 and amino acid 241 to 290. In contrast, fragments F1 (from amino acid 1 to 50) and F3 (amino acid 81 to 130) was recognized by only 3 and 1 antisera out of the 39 samples, respectively (considering signals greater than 10 % of rVP3 O.D. values). Fragment F4 (from amino acid 121 to 170) was recognized by 6 samples. Fragments F5 (residues 161 to 210) and F6 (residues 201 to 250) were detected by several samples (23 recognized F5 and 14, F6), mainly those with higher rVP3 O.D. values. In these cases, at least one of these 2 fragments could be recognized.

To evaluate the repeatability of these assessments, one sample was tested several times and the results are exhibit in Additional file 5.

## Discussion

The main strategy to control IBDV is to vaccinate animals (or the breeding hens) with particular strains of the virus and assess their immunological status using commercial ELISA kits that are sensitized with proteins or whole virion from the same vaccine strain used [40, 41]. However, the kits available may not be the most appropriate ones to evaluate nonvaccinated chickens in other regions of the globe, where different virus strains circulate. There are reports showing the phylogenetic diversity of strains and the concentration of Brazilian isolates in specific clusters [25, 26, 29, 33]. Efforts must be made to investigate the wild type circulating strains and to customize diagnostic devices, in order to cope with the virus variability.

There are very few reports evaluating the performance of diagnostic devices based on recombinant proteins of IBDV [17, 18, 20–22]. We developed an ELISA based on a recombinant VP3 to assess the immunological status of our cohort of vaccinated and nonvaccinated chickens from Paraná State, Brazil. The chosen VP3 sequence to be expressed was from a vaccine strain since the panel of antisera available to be used was extracted from vaccinated chickens. Our recombinant VP3 ELISA showed absolute concordance with the commercial ELISA used as a standard. This good performance of the rVP3 ELISA was also reported by others [20, 22, 24].

Despite the agreement between both ELISA results (our rVP3 and the commercial ELISA), the correlation of O.D. values between them was low (R^2^ = 0.24). Martínez-Torrecuadrada and coworkers [18] also compared their results with commercial tests and showed a high correlation when a recombinant VP2 precursor was used in the ELISA, contrasting the lower correlation with a recombinant VP3 test (R^2^ < 0.4). This low concordance can be reflection of the antigen used to sensitize the plates and the balance of anti-VP2 and anti-VP3 antibodies in the antiserum of each animal [17]. The O.D. signal of the commercial ELISA is a consequence of the binding of antibodies against VP2 and VP3, since the whole virion is used as antigen, and a rVP3 ELISA assesses only anti-VP3 antibodies.

Interestingly, when we tested antisera from nonvaccinated animals (and those of unknown vaccination status) with the rVP3 ELISA and the commercial ELISA, the results were conflicting. The commercial ELISA indicated one positive and eight negative samples, but all those samples were positive with the rVP3 test. Two factors must be considered in this case. First, as already mentioned, the specificity of antibodies detected by the two tests. The recombinant VP3 ELISA detects only anti-VP3 (VP3 being a conserved protein among the strains), while the commercial test detects anti-VP3 and anti-VP2 antibodies (that recognize a very variable protein). Second, the antibodies present in the nonvaccinated animals could have been generated against a different strain from that used in both ELISAs. As we do not know the balance of circulating anti-VP2 and anti-VP3 antibodies, an animal with a higher titer of anti-VP2 could not recognize the commercial ELISA VP2 of a different strain, and the amount of anti-VP3 could not be sufficient to be detected by the antigen in a viral lysate. The rVP3 ELISA, which enhances the capture of anti-VP3 antibodies, even from a different strain since the protein is more conserved, showed that animals from domestic creators came into contact with the IBD virus. The rVP3 ELISA must still be evaluated using antibody panels for other diseases in chickens to rule out the possibility of cross-reactions and false positive results. However, VP3 is a protein present only in birnavirus, and no other virus of this family infects chickens besides IBDV. Based on the negative antisera used in our tests, it was discarded the presence of *E. coli* contaminants in the rVP3 extract that could be being recognized by chicken antisera.

This result reinforces the need to analyze the circulating strains to develop a customized diagnostic device, and special attention should be paid to the nonvaccinated chickens that are domestically maintained in Brazil for the families’ own consumption (both poultry and eggs) without proper sanitation controls. Organic agriculture, which favors animal breeding free of medicines and vaccines, is becoming stronger each day in Brazil. IBDV is a very contagious virus [42, 43], and it would not be unusual if those chickens could be infected.

Our rVP3 was able to assess the immunological status of animals inoculated with inactivated or live attenuated vaccines. If the whole virus is used in the vaccine, our rVP3 test will not differentiate anti-VP3 antibodies resulting from vaccination or wild virus infection, but if it is used a recombinant vaccine based on the VP2 protein, such as Vaxxitek HVT + IBD [44] or fp-IBD1 [45], a suspicious infection in the flock could be detected using the rVP3 ELISA, since the vaccinated animals will present anti-VP2 but not anti-VP3 antibodies.

Epitopes of VP2 and VP3, the most immunogenic virus proteins, have been characterized [11, 13, 14, 19, 35–38, 46–48]. Most of those studies used monoclonal antibodies produced by hybridomas generated from mouse B cells. Monoclonal antibodies are produced from one B lymphocyte clone; hence, the plurality of epitopes along a protein is not assessed. Further, individual animals have distinct genetic backgrounds, and the variability of immune system genes can direct the response to different sets of immunodominant epitopes. If this variability occurs in different animals of one species, it is more exacerbated in animals from different species (*M. musculus* vs. *G. gallus*). Therefore, the epitopes identified by monoclonal antibodies are not necessarily the target of antibodies in a natural infection.

To overcome the limitations of monoclonal antibodies to identify epitopes relevant in the infection process, we analyzed the reactivities of different regions of VP3 against polyclonal antisera from chickens in Brazil. We tested chicken antisera positive for IBDV in an ELISA sensitized with peptides of 130 amino acids covering the full length of the VP3 protein. All the tested samples recognized all fragments. However, the N-terminal (FA) and C-terminal (FP) fragments showed a greater reactivity than the middle fragment (FM). Based on their overlapping, this result could be a suggestion that the most reactive parts of the molecule would be localized between amino acids 1 to 80 and 210 to 290.

To define more precisely the region of VP3 that could be recognized by the chicken antibodies, we divided the molecule into 7 fragments. Although each antiserum sample shows a specific pattern of fragment recognition, it is possible to identify some characteristics. The F4 fragment was recognized by few samples and fragments F1 and F3 were poorly recognized by all antisera. Fragments encompassing amino acids 41 to 90 (F2) and 241 to 290 (F7) were recognized by most antisera. Fragments F5 and F6 (amino acids 161 to 250) were more often recognized after F2 and F7. F5 to F7 span the 105 amino acids of the C-terminal region of VP3, rich in charged amino acids that confer the hydrophilic nature and antigenic properties of this portion of the molecule [49]. Nevertheless, none of the fragments showed to be immunodominant in all antiserum samples.

To our knowledge, our work is the first one to map VP3 epitopes using chicken antibodies. In the face of our results, although the region from amino acids 41 to 90 (F2) and the last 50 amino acids (F7) of VP3 seemed to be a common target to antibodies, some samples such as G6, ME219, ME275 and NC showed greater affinity for other regions. Therefore, it is premature to choose any region of the molecule as one containing a universal epitope to be used in a diagnostic device, at least in the format developed in this work (as a fusion to GFP). The production of the full-length recombinant VP3 was shown to be feasible and inexpensive and would circumvent the situation where an animal could develop an idiosyncratic immune response.

## Conclusions

The ELISA based on the conserved VP3 protein was shown to be suitable to detect infected chickens by IBDV in regions around the globe where the wild strains are different from the ones used to produce the recombinant proteins. In addition, in an effort to detect immunogenic VP3 epitopes, our work was the first to identify several regions recognized by chicken antibodies. Nevertheless, their use for diagnosis of IBD in chickens needs to be further evaluated. The production of the full-length recombinant VP3 still seems to be the better strategy to include all VP3 epitopes that can be recognized by an animal.

## Methods

### Antisera

Antisera from 107 animals were analyzed in this work. Commercial producers provided samples from 35 adult vaccinated chickens (MSD, Merck and Meriel – live attenuated and killed vaccines combinations) and 44 one-day-old chicks whose mothers were vaccinated against IBDV (Hipragumboro – live attenuated vaccine). Seven antisera from nonvaccinated animals and 2 from unknown vaccination status animals were obtained from domestic producers. IBDV-free chicken antisera from 19 animals were supplied by Federal University of Paraná. The vaccination scheme consisted of several doses of attenuated virus vaccine via ocular and inactivated virus vaccine via intramuscular administration.

### VP3 sequence and cloning

A VP3 sequence (GenBank accession number ABI52864.1) was codon-optimized for expression in *E. coli* and purchased from GenScript (New Jersey, USA). The VP3 gene was subcloned into the 6×His tag expression vector pET28a (Novagen) using *BamH* I and *Xho* I restriction endonucleases. Overlapping gene fragments varying in size (150 or 390 bp) and covering the full-length VP3 sequence were PCR-amplified (Fig. 1). The larger VP3 gene fragments (FA, FM and FP, 390 bp) were inserted into pET28a, whereas the shorter VP3 fragments (F1 to F7, 150 bp) were inserted into pET28a-GFP, between *EcoR* I and *Xho* I restriction sites. The pET28a-GFP was constructed by inserting the green fluorescent protein gene, which was PCR-amplified from pEGFP-C3 (Clontech Laboratories, CA, USA), into the *BamH* I site on pET28a (upstream of the *EcoR* I and *Xho* I sites). The *E. coli* DH5α strain was used to propagate the plasmids. The oligonucleotides used in this work are shown in Additional file 6. All constructed plasmids were sequenced to verify the absence of mutations and the correct in-frame insertion of amplicons.

### Recombinant VP3 production

The full-length VP3 and its fragments were expressed in *E. coli* BL21 (DE3) Star (Novagen) with a 6×His tag at the N-terminus after induction with 0.5 mM isopropyl-β-D-thiogalactopyranoside (IPTG) (Amresco). Recombinant VP3 (rVP3, 37 kDa) was expressed at 37 °C for 4 h and solubilized from inclusion bodies with 2 M urea. Larger and shorter VP3 fragments composed of 130 and 50 amino acids, respectively, were expressed at 20 °C for 18 h. The shorter VP3 fragments were tagged to the C-terminus of GFP generating approximately 37 kDa proteins. All fragments could be recovered from the soluble fraction.

The rVP3 and its fragments were purified by affinity chromatography in a Ni-nitrilotriacetic acid (Ni-NTA) resin (Qiagen) and were eluted with 500 mM imidazole. SDS-PAGE and Western blot were performed to evaluate the steps of the expression and purification procedures using a monoclonal antibody anti-polyhistidine as the primary antibody (Sigma) and anti-mouse IgG conjugated to alkaline phosphatase as the secondary antibody (Sigma). The Western blot detection was performed with nitroblue tetrazolium chloride and 5-bromo-4-chloro-3-indolyl phosphate p-toluidine salt (Promega).

### Enzyme-linked immunosorbent assays

A commercial ELISA kit for IBD was used to assess the antibody titer according to manufacturer specifications (product number 99-09260, IDEXX Laboratories, Westbrook, USA).

Plates were coated with 0.25 µg/mL of rVP3 or 2 µg/mL of each VP3 protein fragment. Antisera were diluted 1:500 (for rVP3 as antigen) or 1:100 (for VP3 fragments as antigens), and anti-chicken IgG conjugated to peroxidase (Sigma) was diluted 1:40,000 (for rVP3) or 1:10,000 (for VP3 fragments). The absorbance value was measured at 450 nm. The GFP signal was subtracted from all shorter fragment signals. The cut-off of rVP3 assays was established using a ROC curve performed using MedCalc for Windows, version 16.4.3 (MedCalc Software, Ostend, Belgium). Accuracy measures were evaluated for sensitivity and specificity, comparing the recombinant test performance to a commercial ELISA. 

## Supplementary Information

**Additional file 1.** Recombinant VP3 ELISA optimization. (A) 1:10,000 and (B) 1:40,000 secondary antibodiy dilutions. Negative control: 1; antisera samples: 2 to 8; antisera dilutions: 1:100 (blue bars) and 1:500 (orange bars); antigen concentrations: 0.25, 0.5, 1.0 and 2.0 μg/mL; gray bars: commercial ELISA.

**Additional file 2.** Evaluation of the rVP3 ELISA. (A) rVP3 ELISA A/P values of samples considered negative (left) and positive (right) by the commercial test. The horizontal line represents the cut-off 0.0496. (B) Correlation (R^2^=0.24) between commercial ELISA (y-axis) and rVP3 ELISA (x-axis) O.D. values. A/P = sample O.D. mean – negative control O.D. mean/ positive control O.D. mean – negative control O.D. mean.

**Additional file 3.** O.D. values of larger fragments. O.D. average values of 39 antisera samples tested by ELISA sensitized with FA, FM, FP fragments and rVP3.

**Additional file 4.** O.D. values of shorter fragments. Description of data: (A) O.D. average values of 39 antisera samples tested by ELISA sensitized with F1, F2, F3, F4, F5, F6, F7 fragments, GFP and rVP3. (B) Optical density average values of 39 antisera samples tested by ELISA subtracting O.D. of GFP from F1, F2, F3, F4, F5, F6, F7 fragments.

**Additional file 5.** Repeatability test using ME93 antisera tested in different plates and different days. (A) O.D. average values of ME93 antisera tested by ELISA sensitized with FA, FM, FP fragments and rVP3. (B) O.D. average values of ME93 antisera tested by ELISA sensitized F1, F2, F3, F4, F5, F6, F7, GFP and rVP3. (C) Percentage of O.D. average values of ME93 antisera tested by ELISA sensitized with FA, FM and FP related to rVP3. (D) Percentage of O.D. average values of ME93 antisera tested by ELISA sensitized with F1, F2, F3, F4, F5, F6 and F7 related to rVP3; Percentages inferior to 10% were symbolized as "-"; GFP value of subtracted from fragment values.

**Additional file 6.** Oligonucleotides for PCR assays based on the codon-optimized VP3 sequence. In bold are the targets for restriction endonucleases: *Xho* I - CTCGAG and *EcoR* I – GAATTC.

## Data Availability

Data will be available directly from the authors if required (daniela.pavoni@fiocruz.br).

## Abbreviations

IBD: Infectious bursal disease
IBDV: Infectious bursal disease virus
rVP3: Recombinant VP3
ELISA: Enzyme-linked immunosorbent assay
bp: Base pairs
PCR: Polymerase chain reaction
GFP: Green fluorescent protein
ROC: Receiver operating characteristic
O.D.: Optical density
