# Supplementary figures and images for: Assessing the antigenicity of different VP3 regions of infectious bursal disease virus in chickens from South Brazil
Source: BMC Vet Res. 2021 Jul 30;17:259. doi: 10.1186/s12917-021-02956-0 (PMC8325195; doi:10.1186/s12917-021-02956-0)

**A**


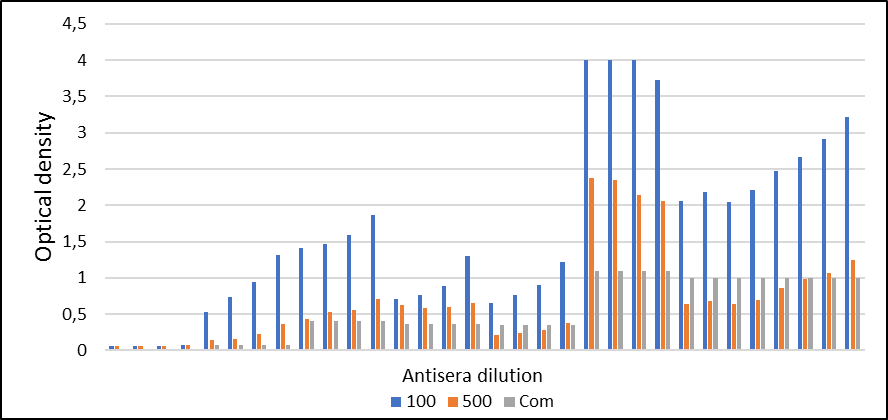


**B**


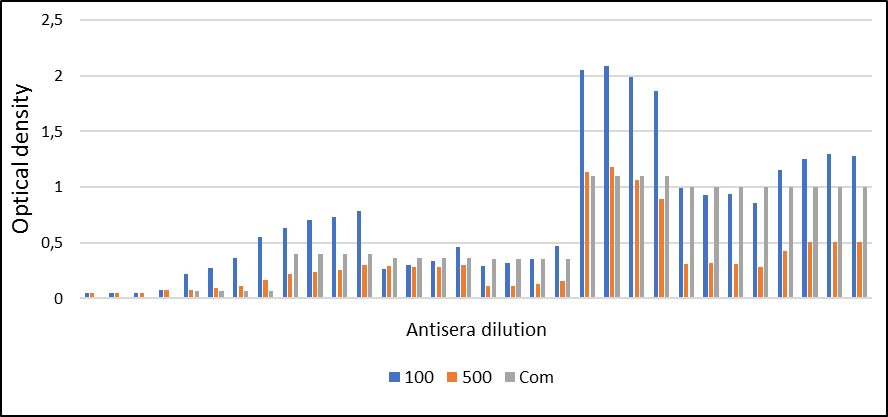

Supplement: Supplementary file 1 — Additional file 1. Recombinant VP3 ELISA optimization. (A) 1:10,000 and (B) 1:40,000 secondary antibodiy dilutions. Negative control: 1; antisera samples: 2 to 8; antisera dilutions: 1:100 (blue bars) and 1:500 (orange bars); antigen concentrations: 0.25, 0.5, 1.0 and 2.0 μg/mL; gray bars: commercial ELISA. [file 12917_2021_2956_MOESM1_ESM.docx]

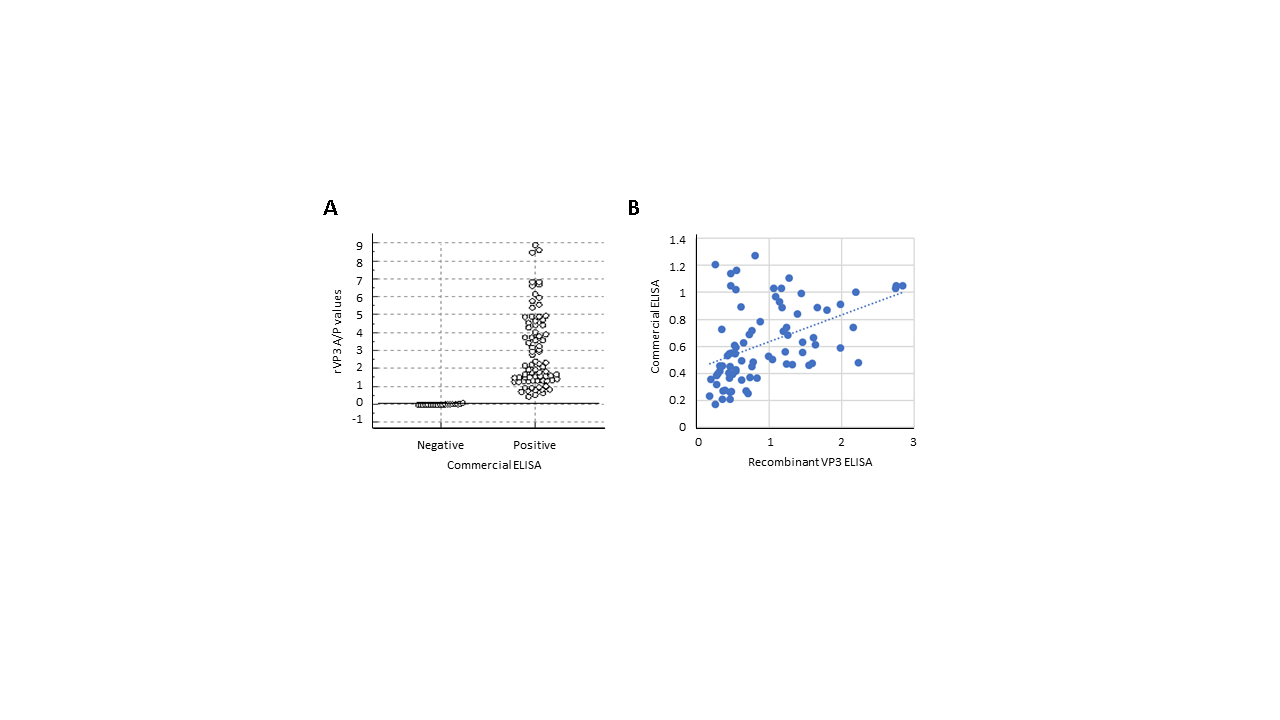

Supplement: Supplementary file 2 — Additional file 2. Evaluation of the rVP3 ELISA. (A) rVP3 ELISA A/P values of samples considered negative (left) and positive (right) by the commercial test. The horizontal line represents the cut-off 0.0496. (B) Correlation (R2=0.24) between commercial ELISA (y-axis) and rVP3 ELISA (x-axis) O.D. values. A/P = sample O.D. mean – negative control O.D. mean/ positive control O.D. mean – negative control O.D. mean. [file 12917_2021_2956_MOESM2_ESM.docx]
