# Supplementary material for: Assessing the antigenicity of different VP3 regions of infectious bursal disease virus in chickens from South Brazil
Source: BMC Vet Res. 2021 Jul 30;17:259. doi: 10.1186/s12917-021-02956-0 (PMC8325195; doi:10.1186/s12917-021-02956-0)
Supplement: Supplementary file 6 — Additional file 6. Oligonucleotides for PCR assays based on the codon-optimized VP3 sequence. In bold are the targets for restriction endonucleases: Xho I - CTCGAG and EcoR I – GAATTC. [file 12917_2021_2956_MOESM6_ESM.docx]

| **Primers** | **Sequence 5' - 3'** |
| --- | --- |
| F1F | CCC**GAATTC**ATGTTTCCGCATAACCCGCGT |
| F2F | CCC**GAATTCC**CGGAACTGGAATCTGCCGTC |
| F3F | CCC**GAATTC**AACTTTGCGCTGTCGGACCCG |
| F4F | CCC**GAATTCC**GTGGTCCGACCCCGGAAGAA |
| F5F | CCC**GAATTC**CCGTCCCCGGGTCAGCTGAAG |
| F6F | CCC**GAATTC**CGTGCCGCAACGAGTATCTAT |
| F7F | CCC**GAATTC**AAGGACCTGCTGCTGACCGCT |
| F1R | CCC**CTCGAG**TTACATTGCACGGACGGCAGA |
| F2R | CCC**CTCGAG**TTACATTGCACGGACGGCAGA |
| F3R | CCC**CTCGAG**TTACATTGCACGGACGGCAGA |
| F4R | CCC**CTCGAG**TTACATTGCACGGACGGCAGA |
| F5R | CCC**CTCGAG**TTACATTGCACGGACGGCAGA |
| F6R | CCC**CTCGAG**TTACATTGCACGGACGGCAGA |
| F7R | CCC**CTCGAG**TTACATTGCACGGACGGCAGA |
